# Under-Replicated DNA: The Byproduct of Large Genomes?

**DOI:** 10.3390/cancers12102764

**Published:** 2020-09-25

**Authors:** Agustina P. Bertolin, Jean-Sébastien Hoffmann, Vanesa Gottifredi

**Affiliations:** 1 Chromosome Replication Laboratory, The Francis Crick Institute, 1 Midland Road, London NW1 1AT, UK; 2 Laboratoire D’Excellence Toulouse Cancer (TOUCAN), Laboratoire de Pathologie, Institut Universitaire du Cancer-Toulouse, Oncopole, 1 avenue Irène-Joliot-Curie, 31059 Toulouse Cedex, France; jean-sebastien.hoffmann@inserm.fr; 3 Fundación Instituto Leloir, Instituto de Investigaciones Bioquímicas de Buenos Aires, Consejo Nacional de Investigaciones Científicas y Técnicas, C1405 BWE Buenos Aires, Argentina; vgottifredi@leloir.org.ar

**Keywords:** DNA replication stress, common fragile sites (CFS), double fork stalling (DFS), under-replicated DNA (UR-DNA), mitotic DNA synthesis (MiDAS), 53BP1, RAD52, break-induced repair (BIR), genomic instability

## Abstract

**Simple Summary:**

Higher eukaryotic cells frequently enter mitosis with a certain load of under-replicated DNA, also referred to as unreplicated DNA, due to incomplete genomic DNA replication during the previous S phase. Double replication fork stalling events, when two converging forks irreversibly stall with no replication origin in between them, seem to be one of the major drivers of incomplete genomic replication in S phase. Genome stability is yet maintained in the vast majority of cells implying that cells must possess dedicated post-replicative mechanisms that allow for faithful repair of these seemingly unavoidable errors. Here, we provide a comprehensive overview of the mechanisms or events that cause, regulate and repair under-replicated DNA in eukaryotic cells.

**Abstract:**

In this review, we provide an overview of how proliferating eukaryotic cells overcome one of the main threats to genome stability: incomplete genomic DNA replication during S phase. We discuss why it is currently accepted that double fork stalling (DFS) events are unavoidable events in higher eukaryotes with large genomes and which responses have evolved to cope with its main consequence: the presence of under-replicated DNA (UR-DNA) outside S phase. Particular emphasis is placed on the processes that constrain the detrimental effects of UR-DNA. We discuss how mitotic DNA synthesis (MiDAS), mitotic end joining events and 53BP1 nuclear bodies (53BP1-NBs) deal with such specific S phase DNA replication remnants during the subsequent phases of the cell cycle.

## 1. Eukaryotic Genome Replication Challenges

Before each cell division, genomes must be entirely and faithfully duplicated. In bacteria, DNA replication commonly initiates at a single, defined location -origin- on a circular chromosome. As a consequence, the time required to duplicate a bacterial chromosome is proportional to its size. In contrast, eukaryotic replication, whilst slower, initiates at multiple origins distributed on multiple chromosomes [1]. The time required to replicate an entire genome is therefore no longer constrained by the genome size but by the distance between origins. This organization allows the fast replication of large amounts of DNA and has probably been decisive in enabling both the evolution of eukaryotes and, in particular, the acquisition of multicellularity, as both require genomes to greatly increase in size and complexity [2]. However, the presence of multiple origins on multiple chromosomes also presents a potential logistical nightmare. Stringent mechanisms are required to coordinate the usage of these origins to ensure the complete and timely replication of the entire genome. No portion of the genome must be replicated more than once, and no portion must be left under-replicated. 

## 2. Re-Replication Events Are Rare

**Figure 1:**
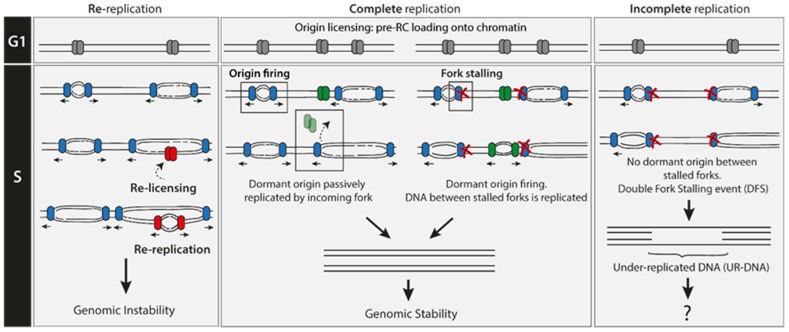
DNA replication must take place once per cell cycle. Such a scenario is represented by the middle panel. No genomic regions should be replicated more than once (left panel), and no regions should be left under-replicated (right panel).

Re-replication occurs when, in a single cell cycle, a portion of an already synthesized DNA is used as an origin of replication and is replicated again (the region is then relicensed and reactivated/fired, Figure 1). Re-replication leads to single-strand DNA (ssDNA) and DNA double-strand breaks (DSBs) formation [3,4]. Origin reactivation has been shown to be a driver of gene amplification, copy number variation, and aberrant chromosome segregation in yeast [5,6]. In mammalian cells, it causes chromosomal breaks, activation of the DNA damage response and genomic instability, ultimately promoting cell death or oncogenesis [7,8,9,10]. Unscheduled re-replication should be differentiated from both ‘scheduled genome re-duplication’ and ‘developmentally programmed re-replication’. Unscheduled re-replication is an aberrant phenomenon associated with genome instability whereas the latter two are highly conserved processes in evolution and are implemented as a form of growth by multiple animal and plant cell types that perform specialized functions [11,12,13].

Prevention of unscheduled re-replication is accomplished in every cell cycle with a simple, but elegant, process that involves two non-overlapping steps: Origin licensing and Origin activation [1,2]. Origin licensing only occurs during telophase and G1 when the activity of S phase cyclin-dependent kinases (CDKs) is low. All the potential replication origins in the genome in a given cell cycle are defined during this step by the loading onto DNA of the pre-replicative complex (pre-RC). The pre-RC comprises the origin recognition complex (ORC), the cell division cycle 6 (CDC6) protein, the CDC10-dependent transcript 1 (CDT1) protein, and the inactive core of the eukaryotic replicative helicase MCM2-7 (minichromosome maintenance complex components 2-7). On the other hand, origin activation or firing takes place during S phase as it requires the combined activity of DBF4-dependent kinase (DDK) and CDK. This step involves the transition from the inactive MCM2-7 complex to the active and processive replicative helicase CMG (CDC45/MCM2-7/GINS) which, together with the recruitment of replication factors, establishes bidirectional DNA synthesis. Outside late M/G1, (i) high-CDK activity mediates licensing inhibition, (ii) Geminin-dependent inactivation of Cdt1 and (iii) specific proteasomal degradation of licensing factors, prevent the relicensing and therefore origin reactivation [14]. The temporal uncoupling between origin licensing and origin activation ensures that each origin will fire once, and only once, in each cell cycle: pre-RCs assemble under conditions that do not allow origin initiation, and origin initiation does not occur until conditions are no longer permissive for pre-RC assembly [15]. Moreover, re-replication is readily detected by the S phase checkpoint and cells with intact checkpoint pathways are able to hinder further re-replication and halt the cell cycle or induce cell death, thus minimizing its detrimental effects [4,16]. Such an active and multi-layered re-replication prevention network is robust, and accordingly, it is highly unlikely that re-replication events happen in a normal cell cycle. Consistent with this idea, re-replication can be significantly detected only in cells with severe dysregulation of the licensing system, and in particular, in cells where both licensing and checkpoint pathways are compromised, such as cancer cells [4,17,18,19]. However, as will be discussed in the next section, the possibility to face under-replication during an unperturbed S phase is much higher.

## 3. Under-Replication Events Are Frequent

### 3.1. Double Fork Stalling (DFS) Events as the Main Source of UR-DNA

During DNA replication elongation in S phase, forks can arrest when they encounter obstacles, such as non-B structured DNA, DNA lesions, transcribing RNA polymerases, DNA breaks or tightly bound protein–DNA complexes [20]. When prolonged or irreversible fork arrest occurs, the converging fork from a neighboring origin can compensate by replicating all of the DNA up to the arrested fork. However, if two converging replication forks stall and there is no licensed origin in between them, a double fork stalling (DFS) event occurs, and the replication of this stretch of intervening DNA has a high chance of being compromised (Figure 1). The main consequence of a DFS event is the generation of under-replicated parental DNA (UR-DNA; also called ‘unreplicated DNA’) and, in some cases, the persistence of this UR-DNA even after the end of S phase. Therefore, the inability to license new origins after the G1/S transition provides a robust aversion mechanism for the re-replication problem but at the same time generates a scenario that favors DFS formation. Pre-RCs are loaded onto DNA in a 3- to 10-fold excess over origins that actually fire during a given S phase [14]. This surplus of licensed origins that remains inactive unless replicative stress happens, named ‘dormant origins’, may have evolved as a solution to this problem, avoiding in that way the more energetically-demanding scenario of increasing the number of active replication forks [14,21]. In fact, experimental modulation of the number of licensed origins in a given G1 phase (by depleting or overexpressing licensing factors), correlates with the respective increase or decrease in markers of DFS in G1 (see Section 8 [22,23]). Such observations establish a strict inverse correlation between the number of licensed origins and the probability of DFS occurrence. 

The probability of accumulating UR-DNA as a consequence of DFS during DNA replication is minimized by the abundance, and positioning, of replication origins but it is *not* cancelled [22,24]. Indeed, genome length seems to determine the *baseline* probability of DFS occurrence [22,24]. This value can be reduced by increasing the number of replication origins and/or changing their distribution along the genome [22]. Consistent with this notion, an evenly spaced distribution of origins across genomes of relatively small size, like the ones in yeasts, can achieve a low and tolerable probability of DFS of a similar magnitude to the chromosome mis-segregation rate [24]. However, as genome size increases, the impact of optimizing replication origin density and/or distribution on DFS probability is significantly reduced [22]. In order to reach a low probability of DFS similar to the one reported in yeast, the density of replication origins in longer genomes should be much higher than the one they actually have [22]. To fulfil that need, origin frequency should reach near-to-saturation levels which might lead to collateral and more undesirable consequences (to name one, it would markedly upregulate the frequency of conflicts between the replication and the transcriptional machinery). Therefore, as genome size changes from megabases (e.g., yeast) to gigabases (e.g., humans), DFSs become increasingly inevitable, although their number during an unperturbed replication cycle remains small (three or less, [22,23,24]). Consequently, a major requirement for genome size expansion in higher eukaryotes has been the evolution of mechanisms that allow effective handling of the DFSs and the spontaneously occurring under-replication generated during each replication cycle.

### 3.2. DNA Loci Which Are Recurrently Prone to Suffer DFS

DFSs can occur at any region of the genome. However, certain DNA loci have an inherent higher propensity for such a replication failure. Such vulnerable DNA loci are characterized by (i) intrinsically hard-to-replicate repetitive sequences which form secondary non-B DNA structures (e.g., centromeres, telomeres, fragile sites); (ii) replication in late S phase, or (iii) paucity of active and dormant origins [25,26]. The chances for DFS accumulation at the end of S phase, though low in unperturbed cells, can be greatly enhanced when cells face replicative stress. A great proportion of DFS events occurs at DNA regions known as common fragile sites (CFSs, [27,28]). CFSs are specific DNA loci prone to exhibit chromosome instability manifested as single-chromatid gaps, breaks and constrictions on metaphase chromosomes after experiencing replication stress in the previous S-phase (as for example, exposure to the DNA polymerase inhibitor aphidicolin -APH- [29]). The current model posits that CFS fragility is mainly determined by three CFS features: (i) late S-phase replication, (ii) paucity of replication origins and (iii) active transcription due to enrichment in large transcription units [25,27,30,31]. Moreover, continuous transcription of CFS region throughout S phase could also affect origin availability as it could promote premature eviction of pre-RCs from chromatin preventing their utilization as replication origins [30,31]. 

Interest in fragile sites rose sharply in the last two decades because of its strong association with neurological disorders, and with genomic instability, copy number variation (CNV) and recurrent genomic rearrangements in human cancers [25,32,33,34,35]. Given their instability and involvement in the development of certain diseases, it is notable that natural selection has not eliminated them. On the contrary, these replication-stress-sensitive loci are conserved throughout mammalian evolution and this may also concern lower eukaryotes, including yeast [36]. The conservation of such apparently disadvantageous loci suggests the existence of a yet undiscovered biologically relevant function for CFSs [29,37,38]. 

Given its landscape, the eukaryotic genome seems to drift into unavoidable incompleteness at the end of S phase. This is the case for a significant percentage of cells, even in the absence of augmented replication stress [22,23]. The inevitability of DFS events, and hence the persistence of UR-DNA regions after S phase finalization, poses an immediate threat to genomic stability and cell viability. As will be discussed in Section 4, such UR-DNA regions are not transduced into signals that prevent the G2/M transition. Nevertheless, as discussed in Section 5, Section 6, Section 7, Section 8, Section 9 and Section 10, eukaryotic cells have evolved to efficiently handle such a stressful situation.

### 3.3. Faulty DNA Replication Termination as a Potential Source of UR-DNA

Genomic DNA replication can be mechanistically divided into three distinct phases: Initiation, elongation and termination. Termination occurs when two forks from neighboring replication origins converge and the duplication of the remaining parental DNA between those forks is completed [39]. Termination events are highly abundant. It has been calculated that nearly 50,000 termination events occur in a typical S phase of mammalian cells [40]. Nevertheless, unlike initiation and elongation, which have been widely studied in the last decades, little is known about the process of termination, particularly in eukaryotic cells. This last stage of DNA replication during S phase comprises at least five unique biochemical processes [39]: (i) the convergence of the two terminating forks where Pif1-family of DNA helicases mediate the resolution of the topological stress caused by the approaching forks [41,42]; (ii) the encounter of the two replisomes [39]; (iii) gap filling between the 3′ end of the leading strand at one fork and the lagging strand of the opposing fork [43]; (iv) replisome dissociation from DNA, where the key regulated step is the ubiquitylation of the CMG component MCM7 promoting CMG unloading from DNA [39,44,45,46,47,48] and (v) decatenation of the sister chromatids, product of complete DNA replication. As a consequence of the complex series of events required for proper termination to occur there is significant potential for deleterious consequences. Terminating forks will certainly result in genomic instability if not resolved properly. In *Escherichia coli*, chromosome over-replication, deletions, and other DNA rearrangements are associated with defective replication termination [49,50,51,52,53,54,55]. Indeed, the termination region of the *E. coli* chromosome is considered a recombination hotspot [55]. Currently, we have limited experimental data regarding the consequences of termination failure in eukaryotic cells, nonetheless, it can certainly be inferred that UR-DNA regions may accumulate, and genomic instability may rise if termination events are dysregulated. 

## 4. How Under-Replicated DNA Escapes Detection by the S-Phase Checkpoint? 

Cells that have not fully replicated their genomic DNA are found to enter mitosis [28,56,57]. It is surprising that such UR-DNA is not detected by the checkpoint machinery. UR-DNA becomes evident to the DNA damage response (DDR) only when cells attempt to condense their chromatin in very late G2/early mitosis [57]. This scenario is in sharp contrast with the strong DDR-activating effect of a few DSBs [58,59]. Such a difference could be explained in part by which DDR pathway is activated in each case. G2-M checkpoint arrest can be initiated by the ATM (Ataxia telangiectasia mutated)/CHK2 (checkpoint protein 2) or the ATR (ataxia telangiectasia related)/CHK1 (checkpoint protein 1) pathway in higher eukaryotes. DNA ends in DSBs activate the ATM/CHK2 route while ssDNA present in UR-DNA regions triggers ATR/CHK1. Under-replicated regions resulting from DFS processing may be infrequent and unable to build up sufficient ATR signal to initiate a global response. Alternatively, UR-DNA regions could be actively prevented from inducing G2-M arrest. The reason why UR-DNA escapes checkpoint detection prompting cells to enter mitosis is not known and warrants further research. 

Recently, a different model for cell cycle progression has been proposed, whereby DNA replication actively controls a brake to mitotic entry by continuously restricting CDK1 and PLK1 activity [60,61]. Once DNA replication stops, this brake is released, and mitosis can occur. Therefore, mitotic entry is driven by the ‘absence of ongoing DNA replication’ rather than by ‘DNA replication completion’. This model aids understanding of why the checkpoint sensors may fail to detect DFSs. When DNA replication is not completed due to the existence of DFS events, probably in regions such as CFSs, the irreversible nature of these events creates a scenario of inactive replication which may enable mitotic entry with incompletely replicated DNA. While the ATR/CHK1 pathway probably plays a key role in UR-DNA regulation [60,62,63,64], the mechanistic details related to its role are not fully elucidated. For example, it is unknown how ATR can trigger differential outputs when activated by either ssDNA from ‘ongoing DNA replication’ or ssDNA from DFS events. 

## 5. Cellular Consequences of Incomplete DNA Replication

For some time, it has been commonly held that the primary line of defense against under-replication is: (i) dormant origin activation; and (ii) checkpoint-dependent cell cycle delay which, when coupled, promote replication completion within a given S phase. However, as discussed in previous sections, by itself this is insufficient: the transition from S-phase to mitosis is not as orderly as once thought, and metazoan cells frequently enter mitosis with incompletely replicated or unresolved chromosomes, especially when facing replication stress. Dedicated mechanisms in subsequent cell cycle phases have evolved to deal with the under-replicated remnants of the previous S phase.

## 6. Mitotic DNA Repair

### 6.1. Mitotic DNA Synthesis

**Figure 2:**
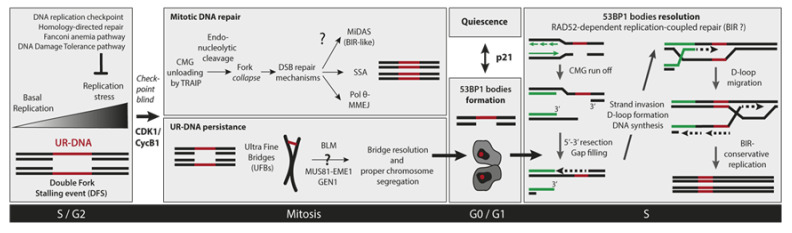
The journey of under-replicated DNA (UR-DNA) across the cell cycle phases. A hypothetical model consistent with the available data describing the fate of the genomic loci with UR-DNA. Low levels of UR-DNA outside S phase, a consequence of DFS events during S phase, seem to be an unavoidable byproduct of gigabase genomes proliferation. Even mild replicative stress greatly increases DFS probabilities. Post-replicative mechanisms can resolve UR-DNA in mitosis or G1/S phase of the next cell cycle. The pathways for mitotic resolution known so far involve either (i) a RAD52 dependent BIR-like synthesis mechanism termed MiDAS, (ii) SSA or (iii) Pol θ-dependent MMEJ. On the other hand, persistent UR-DNA manifests as UFBs in late mitosis and, once resolved by helicases or nucleases action, forms 53BP1-NBs in G1. The presence of mother UR-DNA is a decisive parameter for the proliferation-quiescence decision taken at the M/G1 boundary. The resolution of inherited UR-DNA seems to mainly take place in late S phase through a RAD52-dependent replication-coupled BIR like repair mechanism that is enabled by 53BP1-RIF1 (Rap1-interacting factor 1) coordinated action. Depicted in Figure 2 is a speculative model showing possible DNA transactions for 53BP1-NBs resolution.

Replication stress-induced mitotic DNA synthesis (MiDAS) is a recently discovered DNA repair pathway that occurs after cells have initiated the mitotic prophase. MiDAS buffers the detrimental consequences of replication stress suffered in the previous S phase (Figure 2, [28,56]). MiDAS is not involved in the repair of DNA DSBs or other forms of DNA damage in mitotic cells but mainly deals with DFS-induced UR-DNA at CFSs [27,28,30]. Crucially, not only the replication stress caused by exogenous factors such as APH treatment has been shown to induce MiDAS, but also genetic deficiencies that either slow down replication rate [65] or disturb origin licensing/firing [23]. MiDAS components have been mainly identified based on two characteristics: (i) their requirement for the incorporation of the thymidine analog EdU in mitotic cells previously treated with APH; (ii) their inhibition promotes UFBs in mitosis and 53BP1-NBs in the next G1 (as a consequence of MiDAS failure, see Section 8.1). The non-catalytic subunit of DNA polymerase δ (POLD3) [28], the multifunctional scaffold protein TOPBP1 (DNA Topoisomerase II binding protein 1) [66], the nuclease-scaffold protein SLX4 (Synthetic Lethal of unknown (X) function 4) [28], the structure-specific endonuclease MUS81/EME1 (MMS and UV Sensitive 81/Essential Meiotic Structure-Specific Endonuclease 1) [28,67], the helicase RECQ5 (ATP-dependent DNA helicase Q5) [68], the ssDNA annealing protein RAD52 [69], and the helicase RTEL-1 (Regulator of telomere elongation helicase 1) [70] have all been described as the core components of MiDAS. Surprisingly, this pathway is BRCA2 (breast cancer associated protein 2) and RAD51 independent [69]. While RAD51 is not required for MiDAS activation, its removal from irreversibly stalled replication forks at CFSs is. RAD51 displacement is mediated by the helicase RECQ5 [68]. EdU incorporation in mitotic cells also depends on PLK1 (Polo Like Kinase 1), WAPL (Wings Apart like protein) and SMC2 (Structural maintenance of chromosomes protein 2), implying that MiDAS should occur after or in parallel with the release of sister chromatid arm cohesion [28]. Two recently published papers uncovered one more piece of the MiDAS’s puzzle: how cells switch from conventional S phase DNA replication to mitotic DNA replication [71,72]. The most likely MiDAS substrate is a stretch of UR-DNA which is formed after the processing of a DFS event. These terminally arrested forks and their associated replisomes persist until mitosis and one possibility is that their presence might protect the parental DNA at stalled forks from premature nucleolytic attack. Mitotic replisome disassembly is driven by the polyubiquitination of the CMG component MCM7 by the E3-ubiquitin ligase TRAIP (TRAF-interacting protein) [71,72,73]. TRAIP-directed replisome disassembly is an early requirement for MiDAS. This is potentially due to the exposure of the proper substrate -parental DNA at the junction of the fork- for controlled nuclease activity that leads to fork collapse.

Unlike other DSBs, DSBs at collapsed forks are single ended, with no second end available for classical homologous recombination (HR) repair. This type of DNA damage can be repaired by a HR subpathway called break-induced repair (BIR) which has been mainly studied in yeast [74]. MiDAS mechanistically resembles BIR because it is a HR-based DNA repair pathway used to repair single-ended DSBs that arise at collapsed replication forks following a fork restart event [75]. Both BIR and MiDAS are POLD3- and RAD52-dependent, but classical BIR differs from MiDAS in its RAD51 requirement [69,75,76]. Despite the many open questions which remain to be answered, an attractive model for MiDAS is emerging. High CDK1/CycB mitotic activity seems to initiate the remodeling of irreversibly arrested replication forks at the edges of the UR-DNA. CMG unloading by TRAIP and RECQ5-dependent RAD51 displacement trigger fork collapse, probably driven by SLX4-associated MUS81-EME1 endonuclease. RAD51 removal from stalled forks could be required, not only to allow fork breakage, but also to enable a RAD52-dependent BIR-like process [77]. RAD52 could potentially be recruited to these RAD51-stripped forks and promote MUS81-EME1 localization [69]. The helicase activity of RTEL-1 seems to be strongly required after SLX4 function to enable RAD52 and POLD3 engagement [70]. Like all HR sub-pathways, fork breakage should be followed by end resection to generate a 3′ ssDNA overhang, yet no exonuclease has been reported to participate in MiDAS. This resection will possibly expose a region of microhomology which will then be annealed with a partially single-stranded template DNA by RAD52 promoting DNA Pol δ and POLD3-dependent DNA synthesis. The unwinding of the DNA helix in front of the migrating ‘MiDAS replisome’ should be mediated by helicase activity. Pif1 is the main helicase for yeast BIR [78]; however, the identity of the BIR and MiDAS helicase in human cells remains elusive. It is also important to address the universality of MiDAS, as it was shown that (i) folate stress induces SLX1- and RAD51-dependent (but RAD52 and MUS81 independent) mitotic DNA synthesis at rare fragile sites in human cells [79]; (ii) in non-cancerous human cell lines, APH-induced mitotic DNA synthesis is mainly FANCD2-dependent while RAD52 seems to be dispensable for this process [80].

### 6.2. Mitotic DNA End Joining Events

The Walter lab recently proposed an alternative model for UR-DNA resolution in mitosis, adding one more layer of complexity to UR-DNA management (Figure 2). Using the powerful Xenopus egg-extracts nucleus-free system, they showed that *mitotic* CDK promotes the collapse and breakage of stalled forks [73]. CDK1/CycB1 activates directly or indirectly the E3 ubiquitin ligase TRAIP to ubiquitylate MCM7 CMG subunit, triggering CMG unloading from chromatin by the p97 ATPase [73]. Replisome disassembly initiates, in turn, mitotic fork breakage by an unknown DNA nuclease(s). The newly formed DSB can undergo two main classes of repair events involving end-joining products: (i) single-strand annealing (SSA) and (ii) micro-homology end-joining (MMEJ) [73]. Both often result in DNA rearrangements like deletions, or insertions mediated by POLθ-dependent template switching [73]. Immunodepleting either FAND2 (Fanconi Anemia Complex D2), FANCI (Fanconi Anemia Complex I), SLX4, XPF or MUS81 from extracts did not prevent fork breakage and end-joining events [73]. The authors propose a model in which TRAIP-triggered fork breakage can have a beneficial or detrimental role depending on the amount of stalled replication forks entering mitosis [73]. When few forks are present (e.g., DFSs), CMG unloading triggers a controlled fork breakage by channeling nucleolytic cleavage to the leading strand. This could avoid the generation of acentric and dicentric chromosomes due to random breakage and thus, foster genomic stability [73]. In contrast, when a high number of ongoing or stalled replication forks are present (e.g., premature CDK1 activation in S phase by Wee1 inhibition [81]), massive chromosomal fragmentation occurs, resulting in cell lethality or oncogenic transformation [73].

Of note, there probably is one important mechanistic difference between the aforementioned model and MiDAS which can account in part for the different outputs and it relates to the environment in which replication forks stall. The protocol used for assessing MiDAS reveals forks which have stalled during S phase in the absence of high levels of mitotic CDK. On the other hand, the mitotic end-joining events revealed using Xenopus egg extracts take place in forks that have stalled in a ‘mitotic environment’ characterized by high levels of CDK1/CycB1. 

## 7. UR-DNA Segregation Defects: Ultra-Fine Bridges

Unresolved DNA interlinks that persist into anaphase generate DNA segregation defects that can be broadly classified into two groups: bulky anaphase segregation defects (BADs) and non-chromatinized ultra-fine bridges (UFBs). While BADs can be stained by conventional DNA dyes like DAPI, UFBs cannot [82,83,84]. Although both are described as ‘segregation defects’, it is important to mention that they arise from different types of defects, they are tolerated/resolved by different pathways, and result in dramatically different outcomes regarding genomic instability [82]. BADs are pathological segregation defects that can be connected (chromatin bridges) or not (laggards) to the daughter genomes and they are known to result in aneuploidy or complex genomic rearrangements. Indeed, a big portion of the genomic aberrations seen in cancerous cells can be directly linked to this type of segregation defects [82,83]. 

UFBs are uncondensed and de-chromatinized DNA bridges connecting the two future daughter nuclei and are caused by either: (i) fully replicated catenated DNA; (ii) regions of UR-DNA; or (iii) unresolved recombination intermediates. UFBs are found in unperturbed and stressed conditions; in normal and tumor cells, and are far more prevalent than BADs [82,85]. UFBs result from dysregulated DNA transactions at well-defined chromosomal loci, such as centromeres, telomeres, ribosomal DNA clusters and CFSs. They can be revealed by immunofluorescence staining of a specific set of protein markers. Most if not all UFBs are bound by the PLK1-interacting checkpoint helicase (PICH) as well as the components of the BTRR complex: the BLM (Bloom syndrome protein) helicase and its partners, topoisomerase IIIα (TOPOIII α), RMI1 and RMI2 (RecQ-mediated genome instability 1 and 2) [84,85]. UFBs that arise from UR-CFS, called Fanconi Anemia-associated UFBs, are bound by PICH, BTRR complex, RPA (replication protein A) and in its extremities by FANCD2 and FANCI [82,86,87]. They mainly accumulate after perturbation of DNA replication by APH [86]. UFBs need to disentangle their DNA linkages to grant chromosomal segregation into daughter cells. UFB numbers decrease almost to zero as anaphase progresses suggesting the existence of an active resolution mechanism in anaphase [84,85]. The disjunction of sister chromatids in FA-associated UFBs is accomplished mainly by the ssDNA decatenase activity of the BTRR complex or less frequently, by converting UFBs into DSBs through the action of various nucleases such as MUS81-EME1 or GEN1 (Figure 2, [85,88,89]).

## 8. 53BP1 Nuclear Bodies 

In the following G1, unresolved ‘parental’ UR-DNA from the previous cycle is sequestered in specific G1 nuclear sub-compartments called 53BP1-NBs (Figure 2, [23,88,90]). 53BP1-NBs are distinctive nuclear foci present in G1 and early S phase and, as its name predicts, contain large aggregates of the 53BP1 protein which mark these DNA lesions generated as a consequence of replication stress suffered in the previous cell cycle [90,91]. Remarkably, sister-daughter cells contain 53BP1-NBs which are frequently symmetrical in number and morphology, suggesting that lesions segregate equally to daughter cells [23,90]. Of note, ‘53BP1-NBs’ should not be confused with ‘53BP1 foci’ which are DSB-induced compartments with a much smaller size involved in DSB repair and accumulate exclusively in S phase [92].

53BP1-NBs are ATM-dependent, transcriptionally silent structures that harbor proteins mainly related to DSB signaling: 53BP1, ATM, RNF8 (RING-finger protein 8), RNF168 (RING-finger protein 168), MDC1 (Mediator of DNA Damage Checkpoint 1), γH2AX, BRCA1 (breast cancer associated protein 1), NBS1 (Nijmegen breakage syndrome protein 1, Nibrin) and TOPBP1 [90,91,93]. In contrast, replication stress related proteins seem to be completely absent from these bodies: FANCD2, RPA, ATR, ATRIP (ATR interacting protein), CtIP (C-terminal binding protein 1 interacting protein) and RAD51 [90]. 53BP1-NBs formation in G1 seems to be a universal feature of unperturbed proliferating cells of organisms with gigabase-sized genomes such as human (see Section 3, [23,90]). 53BP-NBs are emerging as the central post-replicative mechanism to deal with the main consequence of DFS events, UR-DNA. Replicative stress during S phase is expected to increase the burden of DFS events in a given cell and in turn, should augment the reliance on 53BP1-NBs for their proper repair. Indeed, 53BP1-NBs greatly increase in frequency in response to mild replicative stress caused by exogenous interference or by particular genetic backgrounds [90,91]. Depletion of proteins involved in DNA replication (e.g., MCM10, Topoisomerase 2A and TOPBP1) [90], DNA replication checkpoint (e.g., ATR, ATRIP and TOPBP1) [90] and classical HR repair pathways (e.g., BRCA2, Partner and localizer of BRCA2 -PALB2-, RAD51, FANCD2 and BLM) [90,94] greatly enhances 53BP1-NBs frequency. Moreover, 53BP1-NBs are enriched in DNA regions with a propensity to suffer DFS such as the CFS regions [67,88,90]. Interestingly, conditions that induce replicative stress and CFS instability (e.g., low/mild-dose APH) foster 53BP1-NB accumulation, while others that induce replicative stress but not CFS instability (e.g., hydroxyurea) are not significantly associated with an increase in 53BP1-NBs [29,91]. Furthermore, as described in Section 3, the number of licensed origins in G1 should modulate the frequency of DFSs experienced by the cell in the following S phase: more licensed origins should decrease DFS events, while less licensed origins should make DFS events more likely to occur. 53BP1-NBs frequency shows the same correlation in those experimental situations: an increase in the number of licensed origins due to overexpression of the licensing factor CDC6 causes a reduction in 53BP1-NBs frequency in the next G1 whereas depletion of the origin licensing factors, MCM5 and CDT1, decreases the number of licensed origins and increases 53BP1-NBs frequency in the next G1 [23,95]. Taken as a whole, these results point towards a model depicting UR-DNA, mainly arising from incomplete replication of CFSs due to DFS events, as the major source of 53BP1-NBs.

### 8.1. 53BP1 NBs: Backup for Insufficient MiDAS or Primary Choice? 

MiDAS and 53BP1-NBs seem to be the main post-replicative mechanisms counteracting the consequences of incomplete replication. Failure to perform MiDAS in the face of replicative stress channels the resolution of the UR-DNA to 53BP1-NBs in the following cell cycle. Indeed, the depletion or chemical inhibition of all the components required for—or related to—MiDAS, have shown to increase 53BP1-NBs in the following G1 phase: SLX4 [28], MUS81 [28,67,88], SMC2 [28,90], replicative polymerases [28], POLD3 [28], TOPBP1 [66], RAD52 [69], RECQ5 [68], TRAIP [71,72], BLM [90] and Polη [56]. Likewise, a considerable amount of UR-DNA due to high replicative stress experienced in the previous S phase could lead to the saturation of MiDAS and prompt its resolution in G1. This could explain why higher doses of replication stress lead to increased MiDAS and increased 53BP1 bodies, both mechanisms working towards the same goal: resolving UR-DNA. Therefore, one could infer that 53BP1 is a compensation mechanism when MiDAS is not functional or when MiDAS is simply not enough. However, one could argue an alternative interpretation: 53BP1-NBs might be indeed the mechanism of choice for dealing with UR-DNA in unperturbed cells and not a mechanism that compensates for insufficient MiDAS. 53BP1-NBs have been observed in unperturbed conditions in every cell studied so far [23,90]. In contrast, in unperturbed cells, MiDAS is low or undetectable, suggesting that MiDAS is chosen mainly when DNA replication is perturbed [27,28]. Moreover, according to [24], 53BP1 preferentially associates with DNA in larger replicons of unperturbed cells which are the regions with the highest probability of a DFS [23]. 53BP1-NBs dependent UR-DNA resolution could be advantageous when compared to MiDAS because mitosis is an exceptionally hazardous stage of the cell cycle in which the genome undergoes rapid and dramatic structural and organizational changes. Hence, cells may choose to tolerate, and not repair, DFSs in mitosis, fixing them in the subsequent cell cycle. The tolerance option could represent a positive choice if one considers that all the UR-DNA mitotic repair options described so far (see Section 6 and Section 10) seem to be highly mutagenic. 

### 8.2. 53BP1-NBs Resolution

The number of 53BP1-NBs present in G1 starts to decline in the early/mid-S phase followed by a rapid dissolution in the late S phase [90,96]. This implies that progression into S phase is required for 53BP1-NBs resolution. Indeed, through the real-time imaging of G1-arrested cells, Spies et al. showed that the majority of 53BP1-NBs are not resolved in G1, suggesting that they cannot be efficiently processed by DNA repair mechanisms that are functional in G1 [96]. They also showed that 53BP1-NBs, prior to their dissolution in late S phase, colocalize with PCNA and EdU, prompting the idea that these bodies need active DNA replication for their repair and subsequent dissolution. New origin firing inhibition greatly reduced the colocalization of EdU with 53BP1 bodies, suggesting that their repair and dissolution are driven by the firing of local replication origins [96]. Using RAD18 and a 53BP1 minimal focus forming region as two independent markers for 53BP1 bodies, they showed that 53BP1 seems not to be fully required for G1 53BP1-NBs formation, but this protein is necessary to enforce replication of these lesions in the late S phase and hence, necessary for their correct dissolution [96]. 53BP1 imposes late replication timing onto 53BP1-NBs by recruiting RIF1 [96]. RIF1 is known for enforcing replication timing by suppressing the premature firing of late origins [97,98]. The obvious question that follows is: which mechanism repairs the UR-DNA in these bodies? RAD52, but not RAD51, transiently but strongly associates with 53BP1-NBs in the very late moments of the dissolution process. Loss of RAD52 results in a marked 53BP1-NBs dissolution defect, denoting the persistence of lesions in the absence of Rad52. This positions RAD52 as an important player in UR-DNA resolution probably channeling the repair to a BIR-like mechanism [96]. 53BP1 and RIF1, together with the shieldin complex, share a common function: they limit DNA-end resection and therefore their action influence the pathway used to repair a given lesion [99]. RIF1 and the shielding complex are not required to recruit RAD52, but to prevent premature unscheduled RAD51 recruitment to the UR-DNA in 53BP1-NBs. Loss of 53BP1, RIF1 or shieldin enables RAD51-mediated repair of 53BP1-NBs-associated UR-DNA generating illegitimate recombination intermediates and fostering chromosome aberrations in the following mitosis. Therefore, 53BP1/RIF1/shieldin presence is required to channel UR-DNA from the previous S phase into a replication-coupled RAD52-mediated repair, restricting both premature origin firing and Rad51 engagement [96]. 

### 8.3. UR-DNA Safeguarding Properties of 53BP1

**Figure 3:**
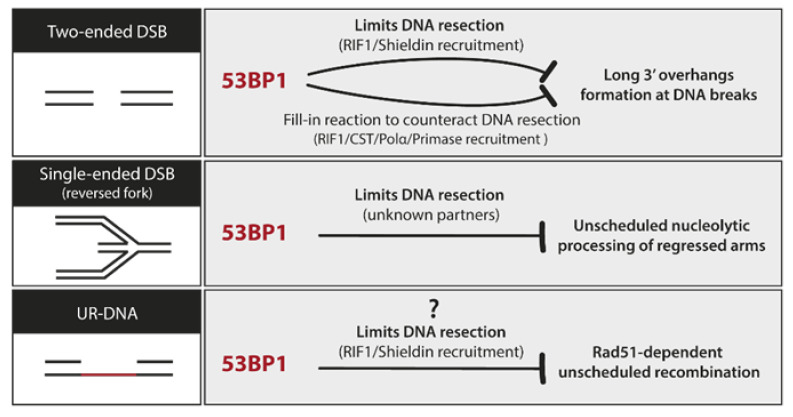
An overview of 53BP1 role in distinct DNA lesions. 53BP1 is a master regulator of the outcome of DNA repair mainly through its ability to control the processing of DNA ends.

53BP1 was originally discovered based on its role as an interactor of the tumor suppressor, p53 [100]. More than 25 years later, the biology of 53BP1 has taken a life of its own. 53BP1 is a large chromatin-binding protein that functions as a molecular scaffold, bridging interactions between damaged chromatin and several effector proteins implicated mainly in DNA lesion processing [99,101,102]. Its role in DSB repair has been studied in much detail. The extent of 5’-3′ nucleolytic digestion of broken DNA ends determines which repair pathway is chosen to process these lesions: No or minimally processed DSB ends are repaired by non-homologous end joining (NHEJ), whereas resected DSB ends are repaired by homology-directed repair (HDR) [102]. 53BP1 is essential for this choice: it limits DNA resection at broken ends, and in some cases even reverses it, greatly increasing the use of NHEJ in certain contexts [101,103]. 53BP1 is not a core NHEJ factor, yet it is critical for both the execution of physiological NHEJ-driven events like immunoglobulin class switching and for the triggering of pathological ones, like the fusion of unprotected telomere ends or the chromosomal aberrations seen in cells deficient in BRCA1 [103,104]. Moreover, 53BP1 is important not only for the NHEJ/HDR pathway choice but also for the choice between different HDR sub-pathways. By preventing hyper-resection of DSB ends, 53BP1 channels DSB repair to RAD51-dependent error-free HR avoiding the highly mutagenic RAD52-dependent SSA [105,106]. SSA is a dangerous HDR choice in regions of the genome with many repetitive sequences, increasing the likelihood of deletions and insertions as the outcome of the repair. Recent evidence shows that 53BP1 can also curb the nascent DNA degradation driven by MRE11 at reversed forks, broadening its role to other types of lesions besides two-ended DSBs (Figure 3, [107,108]). 

An alternative view of 53BP1 is emerging, where its primary biological purpose is to block illegitimate recombination events so as to warrant the fidelity of DNA repair [101]. This is mainly achieved through its ability to limit nucleolytic resection (Figure 3, [101]). The role of 53BP1 in 53BP1-NBs may be another example of this: 53BP1 shields UR-DNA against erosion during G1 and fosters the fidelity of its repair in S phase by suppressing unscheduled recombination [96]. 

## 9. The Biological Significance of Transgenerational Transmission of DNA Damage

We are just beginning to understand the fact that the completion of genome duplication may take more than a single cell division cycle. Devoted post-mitotic repair mechanisms have evolved to counteract this DNA damage and ease its impact on genome stability. 53BP1-NBs are observed in approximately one-fourth of unperturbed G1 cells implying, as discussed in previous sections, the stunning high prevalence of inherited DNA damage, mainly in the form of UR-DNA [23,90,91,109]. Nevertheless, genome stability is preserved in the vast majority of those cells, suggesting that these post-replicative mechanisms are able to accurately resolve most of those lesions. Currently, it is unclear how such extensive amounts of UR-DNA are later duplicated. The Lukas lab shed some light on this question by showing that a percentage of 53BP1-NBs are resolved in the next S phase through a RAD52-dependent replication-coupled repair mechanism (see Section 8.2) [96]. New data from the cell cycle field added another layer of complexity: the amount of inherited DNA damage has a direct impact on the immediate fate of daughter cells [79,80,81]. After each mitosis, actively proliferating cells assess not only the availability of mitogens but also their ‘genome health’ (unresolved lesions still present) and then decide whether to continue proliferating (S phase entry commitment) or to activate a p21-dependent transient cell cycle exit (quiescence/G0 state) [109,110,111]. Daughter cells with one or more 53BP1-NBs tend to enter into a p21-dependent reversible quiescent state. The purpose of this arrest is not fully understood. 53BP1-NBs seem not to be resolved while in a quiescent state as the number of bodies present in a cell that transitions into quiescence and then re-enters the cell cycle does not change [109]. The transient exit from the cell cycle may allow cells to fully prepare for proper DNA damage repair and resolution in the next S phase. Furthermore, the frequency of 53BP1-NBs is associated with an extended G1 duration [111,112]. This result suggests that, although 53BP1-NBs are not resolved in G1, they are sensed in G1 triggering activation of molecular pathways leading to G1 arrest and perhaps setting the scene for their repair during the next S phase. Loss of p53, but not p21, overrides G1 lengthening in this context and allows S phase entry [111,112]. Further research will determine the impact of such transient quiescence state on 53BP1-NBs metabolism and the relevance of such an interplay on the overall genomic stability of daughter cells. 

## 10. The Enigmatic Error-Prone Nature of UR-DNA Repair

As described in the previous sections, post-replicative mechanisms have evolved to cope with the burden of DFS events and their immediate consequence, UR-DNA. However, the error-prone nature of all the mechanisms described so far seems to challenge the genomic stability of cells [113]. 

Mitotic repair of UR-DNA involves at least two pathways: (i) MiDAS (see Section 6.1, [28]) and (ii) DNA end joining events (see Section 6.2, [73]). MiDAS is a form of RAD51-independent BIR that occurs in mitosis. Even though RAD51-independent BIR occurrence has been extensively documented, most of its mechanistic details remain obscure. What we do know is that canonical BIR is a mutagenic and genome rearrangement–prone repair mechanism in yeast and humans [114]. Errors in BIR arise due to improper recombination transactions and to the strong predisposition of the BIR replisome to make misincorporation, frameshift and template-switching errors [74]. Moreover, classical BIR is mainly conservative [115,116]; nevertheless, the nature of BIR synthesis during MiDAS is still a matter of controversy. While initial reports suggested that MiDAS might comprise conservative replication like BIR, as MiDAS foci seem to involve mainly one of the two sister chromatids [69,116], more recent reports suggest that APH-triggered MiDAS is mostly semiconservative [117,118]. A semi-conservative mitotic replication would cause less genetic loss than a conservative one and, therefore, it would be favored in a gigabase genome-context where at least one DFSs in every S phase is very likely to occur [22,23]. Mitotic DNA end joining events, on the other hand, seem to mainly involve SSA and MMEJ-mediated resolution of broken forks [73]. Both are error prone homology-based DSB repair pathways that have been associated with chromosomal rearrangements events [119]. SSA is driven by extensive resection followed by RAD52-mediated homology search between homologous repeats and can lead to deletions of hundreds of kilobases [119]. POLθ, the main polymerase in MMEJ, can perform template-independent strand synthesis, leading to the genomic insertion of random sequences [120]. Indeed, insertions and deletions arising from the dysregulated use of these mechanisms have been associated with the tumorigenesis of homologous recombination deficient cancers [121,122]. Subsequent repair of UR-DNA in the next cell cycle involves the formation and resolution of 53BP1-NBs (see Section 8, [90,96]). 53BP1-NBs resolution is mediated by replication-coupled repair of UR-DNA within 53BP1 bodies. The repair mechanism seems to involve RAD52-dependent and RAD51-independent BIR, as is the case during MiDAS. As mentioned before, BIR DNA synthesis is intrinsically inaccurate due to the unstable nature of its replication intermediates [114]. Further studies are needed to confirm whether BIR is the primary replication-coupled DNA repair option for UR-DNA in 53BP1-NBs, and if so, whether its error-prone nature can be minimized [123].

It is puzzling that cells attempt to complete faulty S phase replication in subsequent cell cycle phases by utilizing mutagenic repair mechanisms that might compromise genome integrity. Further work is required to understand precisely when these error-prone pathways are employed, how these mechanisms are regulated and, notably, how the augmented genomic instability derived from the error-prone nature of these processes is kept in check.

## 11. Clinical Relevance and Concluding Remarks

In recent years, some of our long-standing views in the field of DNA replication have been challenged and even refuted (e.g., ‘Complete genome replication should occur before mitosis’). We now know that cells frequently enter mitosis with a certain load of UR-DNA. While UR-DNA is certainly a challenge to the DNA damage response, it can also harbor a potent and unique role in the transgenerational signaling between mother and daughter cells. While much more research is needed to fully understand the mechanisms that cause, regulate and repair UR-DNA, we can already grasp its potential relevance for the genesis and the treatment of cancer. 

*UR-DNA as a trigger of oncogenesis during oncogene activation*. Current models posit late S phase replication, origin paucity and persistent transcription as the central drivers of CFS expression (see Section 3). Oncogene activation can induce replicative stress in several ways, including decreased number of licensed origins [124] and greatly increased global transcription [125]. Therefore, oncogenic-induced replicative stress could highly raise the chances of a DFS event in CFS loci, UR-DNA generation and its improper resolution (e.g., CNV induction) [25,126,127]. Indeed, *de novo* CNVs (deletions and amplifications) arise frequently in cancers, usually in large CFS-associated genes [25]. Furthermore, if a tumor suppressor gene is located in a CFS, its inactivation due to oncogene-induced replicative stress could significantly contribute to the cell’s tumorigenic potential. 

*UR-DNA repair as a target in anticancer therapies*. Synthetic lethality refers to cell death as the outcome of the combined inhibition of two genes/pathways while the individual loss of function is nonlethal. Tumor cells usually harbor specific defects in DNA repair pathways and experience high levels of replicative stress. To potentiate their survival, cancer cells activate alternative repair mechanisms that can be targeted by synthetic lethality strategies as they would only be lethal for tumor cells. For example, BRCA1/2 and PARP1 synthetic lethal interaction has been successfully applied as an anticancer therapy [128]. BRCA1/2-deficient tumors, which present increased CFS fragility and 53BP1-NBs frequency [94], might also rely on mitotic UR-DNA repair for survival as they exhibit synergistic genomic instability and synthetic lethality with the additional loss of the MMEJ pathway factor Pol θ [122,129] or RAD52 [130,131,132]. These observations provide a rationale for the development of Pol θ or RAD52 inhibitors that can target BRCA-deficient tumors.

Notably, another genetic background that could be suitable for targeting the UR-DNA repair pathway as an anticancer therapy is p53 deficiency, which represents by far the most frequent genetic alteration in human cancers [133]. Cells with higher-than-average numbers of 53BP1-NBs either enter in a p21-dependent reversible quiescence state or lengthen G1 phase in a p53-dependent p21-independent manner (see Section 9). Although the biological function of these two responses is not fully understood, it is tempting to speculate that p53 deficient tumors could have an altered capacity to resolve 53BP1-NBs and, hence, they could rely on the mitotic repair pathway to resolve UR-DNA.
